# The reach of Spanish-language YouTube videos on physical examinations made by undergraduate medical students

**DOI:** 10.3352/jeehp.2017.14.31

**Published:** 2017-12-19

**Authors:** José M. Ramos-Rincón, Isabel Belinchón-Romero, Francisco Sánchez-Ferrer, Guillermo Martínez-de la Torre, Meggan Harris, Javier Sánchez-Fernández

**Affiliations:** 1 Department of Clinical Medicine, School of Medicine, Sant Joan d’Alacant Campus, Miguel Hernandez University of Elche, Elche, Spain; 2 Department of Pediatrics, School of Medicine, Sant Joan d’Alacant Campus, Miguel Hernandez University of Elche, Elche, Spain; 3 Innovation and Teaching Support Service, Elche Campus, Miguel Hernandez University of Elche, Elche, Spain; 4 Independent Researcher, Valencia, Spain; Hallym University, Korea

**Keywords:** Video recording, Physical examination, Medical education, Learning resources, Clinical skills, Spanish

## Abstract

This study was conducted to evaluate the performance and reach of YouTube videos on physical examinations made by Spanish university students. We analyzed performance metrics for 4 videos on physical examinations in Spanish that were created by medical students at Miguel Hernández University (Elche, Spain) and are available on YouTube, on the following topics: the head and neck (7:30), the cardiovascular system (7:38), the respiratory system (13:54), and the abdomen (11:10). We used the Analytics application offered by the YouTube platform to analyze the reach of the videos from the upload date (February 17, 2015) to July 28, 2017 (2 years, 5 months, and 11 days). The total number of views, length of watch-time, and the mean view duration for the 4 videos were, respectively: 164,403 views (mean, 41,101 views; range, 12,389 to 94,573 views), 425,888 minutes (mean, 106,472 minutes; range, 37,889 to 172,840 minutes), and 2:56 minutes (range, 1:49 to 4:03 minutes). Mexico was the most frequent playback location, followed by Spain, Colombia, and Venezuela. Uruguay, Ecuador, Mexico, and Puerto Rico had the most views per 100,000 population. Spanish-language tutorials are an alternative tool for teaching physical examination skills to students whose first language is not English. The videos were especially popular in Uruguay, Ecuador, and Mexico.

## Introduction

Physical examinations play an integral role in clinical practice despite the phenomenal advances in imaging technology and laboratory medicine over the past several decades [1]. Medical curricula include a strong focus on this core competency [2]. While formal instruction has traditionally been the primary source for learning these clinical skills [3], the Internet is becoming an important complementary resource for up-to-date, globally available information [4].

As the largest Internet video-sharing site, YouTube constitutes a promising learning resource, including for medical education and research [5]. Recent studies have evaluated the platform’s potential to support English-language education on physical examinations for the cardiovascular, respiratory, and nervous systems [6], but there is scant evidence for other globally prominent languages such as Spanish, which is spoken in more than 20 countries of varying income brackets [7]. The aim of this study was to evaluate the performance and reach of video tutorials performed by medical students on physical examinations and uploaded onto YouTube.

## Videos and setting

A small group (N=7) of third-year medical students, in collaboration with 2 undergraduates in the audiovisual communications program, developed 4 videos on physical examinations during the 2013– 2014 academic year at the Miguel Hernández University School of Medicine (Elche, Spain). The tutorials focused on examinations of the head and neck (7:30), the cardiovascular system (7:38), the respiratory system (13:54), and the abdomen (11:10) (Appendix 1). The medical students drafted the script under the supervision of a professor. Two students were featured in each video: one as the patient and the other as a doctor. Meanwhile, undergraduates studying audiovisual communications directed, recorded, and edited the videos, which were published online on February 17, 2015 and were included in the official list of the university’s video productions and embedded on the Integrated Workshop course website.

## Data collection

We extracted the visualization metrics for the period from February 17, 2015 to July 28, 2017 (2 years, 5 months, and 11 days) from the YouTube channel administrator page, using the Analytics application offered by the platform to track the performance of the channel and the videos (YouTube Creator Hub. YouTube partners: guide to your YouTube analytics: https://www.youtube.com/watch?v=AUU9urHAwco&feature=youtu.be). The Analytics report is broken down into 6 cross-linked categories: performance metrics, which monitor the watch-time, views, and revenue; interaction metrics, which show data on how viewers interact with the videos (comments, likes, shares, and tags); the demographics report, which presents information on viewers’ sex, age, and location; the traffic sources report, which shows how viewers find the content (search terms and links from other websites); the devices report, which details the types of devices and operating systems used; and the audience retention report, with indicators related to the average view duration.

## Data analysis

We collected demographic data for viewers in Spanish-speaking countries, according to watch-time per 100,000 inhabitants (using population data from 2017 [https://data.worldbank.org/country]). We also compared the 4 tutorials within the YouTube search engine (Table 1). The Miguel Hernandez University Committee of Project Evaluation (DMC.JRR.01.17) approved the study.

## Performance metrics

**Fig. 1.**
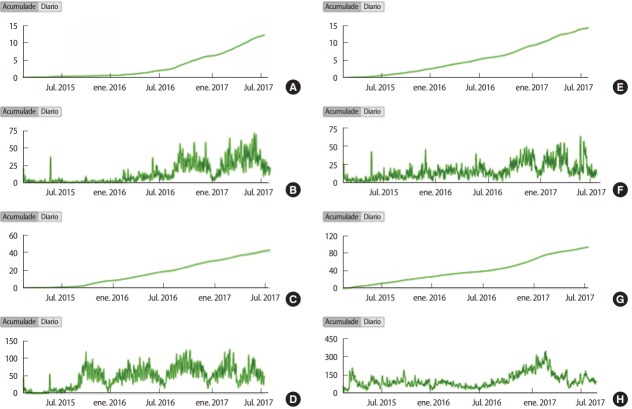
Daily and accumulated views: examination of the head and neck (A, B), examination of the cardiovascular system (C, D), examination of the respiratory system (E, F), and examination of the abdomen (G, H).

The total number of views, length of watch-time, and mean view duration for the 4 videos were, respectively: 164,403 views (mean, 41,101 views; range, 12,389 to 94,573 views), 425,888 minutes (mean, 106,472 minutes; range, 37,889 to 172,840 minutes), and 2:56 minutes (range, 1:49 to 4:03 minutes). The abdominal examination garnered the most views; however, viewers spent slightly more total minutes watching the respiratory system tutorial. The mean watch-time per view was 2:56 minutes (range, 1:49 [16.2% of the total video] to 4:03 minutes [40.1%]). The shorter videos (head and neck and cardiovascular system) retained viewers for more than 35% of the total playback time. Fig. 1 shows the daily and accumulated views for the 4 videos; some show seasonal differences that could correspond to academic study periods. The raw data are available in Supplement 1.

## Traffic sources

The main sources of traffic by views were the “Suggested Video” thumbnails on the YouTube platform, followed by YouTube searches for key terms, and to a lesser extent embedded videos on external websites; 99% of all views took place directly on the YouTube website.

## Demographics

More men watched the videos than women (range, 51% to 77%). The most absolute views took place in Mexico, surpassing Spain and other high-population Latin American countries such as Colombia, Venezuela, Argentina, and Peru (Table 2). Ecuador (116 views per 100,000 population) and Uruguay (114 views per 100,000 population) were among the countries where the tutorials were most popular relative to the population. Spain, Bolivia, and Mexico followed (108, 92, and 90 views per 100,000 population, respectively) (Table 2). The videos were also viewed in Puerto Rico (91 views per 100,000 population) Brazil, the United States, and Portugal. See Appendix 2 for complete data on views and playback locations.

## Interaction

Our videos received 78 to 213 likes and 1 to 19 dislikes. Of the 38 total comments on the 4 tutorials, 19 were directly related to the content or quality of the videos. Seven comments were straightforward statements of support or gratitude, while 12 showed critical engagement with the content. The other 19 comments were noncontent-related (e.g., opinions on students’ appearance or manner).

## YouTube searches

The 4 videos under study consistently appeared among the top 5 retrieved when using Spanish search terms for the different kinds of physical examinations. Among the competing tutorials yielded by the search, one on head and neck examination had been uploaded a year before and had 25,606 views, while another was 6 years old and had 23,505 views. For the cardiac examination, the other videos in the top 5 positions had 92,000 to 124,000 views and were about 4 years old; for the respiratory system, they had 80,000 to 200,000 views and were 2 to 5 years old; and for the abdominal examination, they had 276 to 35,000 views and were 1 to 9 years old.

## Conclusion

The Analytics application is integrated into the YouTube platform, allowing users to monitor the performance of their videos and channels by means of continuously updated indicators and reports [8]. This functionality has enabled studies such as ours.

As reported elsewhere [4-6,8], a number of high-quality tutorials on physical examinations are available in English on YouTube. Specifically investigating the reach of our university’s video productions has illustrated the general relevance and importance of Spanish media in Internet medical education. It is important to promote the creation and use of these tutorials among medical students, particularly in the area of physical examinations.

The most populated Spanish-speaking country, Mexico, also showed the most views; it also ranked highly in views relative to the population, which is consistent with other studies reporting an intense interest in medical education content in Mexico [9]. More men watched the videos than women in our study, in contrast with the demographic make-up of the medical student body in Spain and Latin America, where women tend to outnumber men [9].

The views per 100,000 population were also particularly high in Ecuador and Uruguay, confirming the appeal of educational content and communications technology there [10]. Puerto Rico, a U.S. territory, also viewed the tutorials relatively frequently. Countries with limited Internet access or other communication barriers, such as Cuba, did not appear on the list of playback locations.

Our videos were filmed by and for medical students under faculty supervision. They are not professional productions, but rather an additional teaching tool, which further reinforces their academic focus. Despite the purely academic content, however, half of all comments expressed negative or clearly offensive opinions on students’ appearance or manner, especially but not exclusively in relation to female students. This pattern follows general Internet trends and confirms the widespread presence of online harassment even in scientific Internet fora.

Physical examination tutorials in English are widely available, as a component of the learning materials included with medical textbooks, as content directly uploaded onto YouTube (e.g., as an aid to preparing the objective structured clinical examination, or OSCE [Geekymedics, https://geekymedics.com/about/]), as part of the video library of journals such as the Journal of Visualized Experiments (https://www.jove.com/), or as material produced by medical schools. General physical examination apps have also been developed for medical students and doctors as a tool for practicing medicine. Despite their high quality, oral understanding can be an obstacle for non-native speakers, and some require paid subscriptions. These barriers underscore the importance of making tutorials available in different languages.

The limitations of this study include its exclusive focus on videos published by a single university in Spain. Moreover, we did not analyze the quality of the videos themselves, as has been done elsewhere [1,3,5].

The promising results achieved have encouraged us to continue producing videos of physical examinations with medical students serving as scriptwriters and actors. We are convinced that this exercise further promotes personal responsibility in the learning process.

These Spanish tutorials are an audiovisual teaching alternative to those available in English, constituting a systematic and planned element of structured teaching. They can contribute to achieving learning objectives, reinforce skills taught in other settings, and maintain students’ interest in the field. Both the medical students producing the videos, and the Spanish-speaking medical education community at large, have benefitted from these materials. Further research into the details and the quality of these videos is warranted.

## Figures and Tables

**Table 1.** Characteristics of the videos analyzed in this study and the main performance indicators

Video characteristic			All videos	Examination of the head and neck	Examination of the cardiovascular system	Examination of the respiratory system	Examination of the abdomen
Name in Spanish			-	Exploración de cabeza y cuello	Exploración cardiaca	Exploración torácica y pulmonar	Exploración de abdomen
Length (min)			10:3	7:30	7:38	13:54	11:10
Date of publication			17-2-2015	17-2-2015	17-2-2015	17-2-2015	17-2-2015
Date of analysis			28-7-2017	28-7-2017	28-7-2017	28-7-2017	28-7-2017
Performance metrics							
	Views						
		Watch-time (min)	425,888	37,889	40,194	174,960	172,845
		No. of views	164,403	12,389	14,368	43,073	94,573
		Mean duration of views (min)	2:56	3:03	2:47	4:03	1:49
		Audience retention (mean % of video watched)	29.1	40.1	36.5	29.1	16.2
Interaction metrics (No.)							
	Sharing						
		Shares	501	104	64	189	144
		Videos on playlist	775	106	114	240	315
		Subscriptions	148	19	18	68	43
	Comments						
		Likes	673	93	78	289	213
		Dislikes	27	5	1	2	19
		Written comments	38	3	3	19	13
Traffic sources (%)							
	Source of traffic by watch-time						
		Suggested videos	60.5	41	53	71	77
		YouTube searches	22.5	44	29	17	8.4
		Embedded video	5.5	7.1	6.8	4.0	4.2
		Other	9.5	8.6	11	7.5	11
	Web location by watch-time						
		YouTube platform	99	99	99	99	99
		External website (embedded)	1	0.9	0.6	0.5	1.3
	Type of device						
		Computer	57.1	55.4	58.6	66.5	47.8
		Mobile phone	29.6	30.7	26.8	22.8	38.1
		Tablet	11.2	12.0	12.8	8.6	11.2
		TV	1.7	1.6	1.6	1.7	2.1
		Video game console	0.3	0.2	0.2	0.2	0.5
		Unknown	0.2	0.1	0.1	0.2	0.3
Demographic indicators (%)							
	Views by sex						
		Men	60	51	63	55	72
		Women	40	49	37	46	28

**Table 2.** Views by playback country and per 100,000 population

Variable	Pop. In thousands	N views	% Views	N views/100,000 Pop.
Latin American countries				
Mexico	122,916	111,094	26.09	90.38
Spain	46,491	49,997	11.74	107.54
Colombia	49,067	29,960	7.03	61.06
Venezuela	31,236	23,866	5.60	76.41
Argentina	43,823	22,495	5.28	51.33
Peru	31,660	22,367	5.25	70.65
Ecuador	16,656	19,359	4.55	116.23
Chile	18,286	16,271	3.82	88.98
Bolivia	11,066	10,188	2.39	92.07
Dominican Republic	10,123	4,813	1.13	47.55
Uruguay	3,487	3,963	0.93	113.65
Guatemala	16,896	3,561	0.84	21.08
Honduras	8,795	2,511	0.59	28.55
El Salvador	6,551	2,159	0.51	32.96
Nicaragua	6,361	2,137	0.50	33.60
Costa Rica	4,949	2,011	0.47	40.63
Paraguay	6,905	1,930	0.45	27.95
Panama	3,842	1,062	0.25	27.64
Other countries				
United States	325,318	17,468	4.10	5.37
Brazil	207,012	5,175	1.22	2.50
Puerto Rico	3,405	3,083	0.72	90.54
Portugal	10,265	695	0.16	6.77
Total		425,889		

Pop., population.

## Supplementary materials

Supplement 1. Data file is available from https://doi.org/10.7910/DVN/H5STPK.

Supplement 2. Audio recording of the abstract.

## Appendix 1 List of links

- Tutorials focused on examinations of the head and neck (Vídeo Exploración de Cabeza y Cuello (umh1935 2014-15). URL: https://www.youtube.com/watch?v=bgRuDFPhGkc&feature=youtu.be), the cardiovascular system (Exploración Cardíaca (umh1935 2014-15). URL: https://www.youtube.com/watch?v=ABinhJN2Ccw&feature=youtu.be), the respiratory system (Exploración Torácica y Pulmonar (umh1935 2014-15). URL: https://www.youtube.com/watch?v=8EGZoWeQZXQ&feature=youtu.be.), and the abdomen (Exploración de Abdomen (umh1935 2014-15). URL: https://www.youtube.com/watch?v=wYoUWqAzWqo&feature=youtu.be).

- The official list of the university’s video productions. URL: https://www.youtube.com/playlist?list=PLClKgnzRFYe60dX94diDj0pCEOJO_9wjs.

- Integrated Workshop subject website. URL: http://umh1935.edu.umh.es/material/videos-tutoriales-de-exploracion-y-semiologia.

## Appendix 2. View metrics by tutorial, country, and population

**Table:**

Variable		Pop. in thousands	Examination of the head and neck				Examination of the cardiovascular system				Examination of the respiratory system				Examinations of the abdomen			
			N views (% column)	No.	N views/100,000 pop.	No.	N views (% column)	No.	N views/100,000 pop.	No.	N views (% column)	No.	N views/100,000 pop.	No.	N views (% column)	No.	N views/100,000 pop.	No.
Latin American countries																		
	Mexico	122,916	13,688 (36.1)	1	11.1	1	13,315 (33.1)	1	10.8	3	51,644 (29.5)	1	42.0	5	32,447 (18.8)	1	26.4	4
	Colombia	49,067	3,356 (8.9)	3	6.8	8	1,730 (4.3)	4	3.5	9	17,002 (9.7)	3	34.7	8	7,872 (4.6)	3	16.0	10
	Spain	46,491	4,396 (11.6)	2	9.5	6	8,071 (20.1)	2	17.4	2	18,349 (10.5)	2	39.5	6	19,181 (11.1)	2	41.3	1
	Argentina	43,823	1,825 (4.8)	6	4.2	13	1,431 (3.6)	6	3.3	10	12,051 (6.9)	4	27.5	10	7,188 (4.2)	5	16.4	9
	Peru	31,660	2,102 (5.5)	5	6.6	9	1,549 (3.9)	5	4.9	7	11,979 (6.8)	5	37.8	7	6,737 (3.9)	6	21.3	6
	Venezuela	31,236	3,039 (8.0)	4	9.7	5	3,113 (7.7)	3	10.0	4	10,049 (5.7)	8	32.2	9	7,665 (4.4)	4	24.5	5
	Chile	18,286	979 (2.6)	9	5.4	10	557 (1.4)	10	3.0	12	11,454 (6.5)	6	62.6	1	3,281 (1.9)	8	17.9	8
	Guatemala	16,896	362 (1.0)	11	2.1	17	191 (0.5)	14	1.1	18	2,116 (1.2)	11	12.5	18	892 (0.5)	12	5.3	18
	Ecuador	16,656	1,764 (4.7)	7	10.6	3	1,169 (2.9)	7	7.0	5	10,149 (5.8)	7	60.9	2	6,277 (3.6)	7	37.7	2
	Bolivia	11,066	1,111 (2.9)	8	10	4	584 (1.5)	9	5.3	6	6,153 (3.5)	9	55.6	3	2,340 (1.4)	9	21.1	7
	Dominican Republic	10,123	806 (2.1)	10	8	7	324 (0.8)	11	3.2	11	2,246 (1.3)	10	22.2	11	1,437 (0.8)	10	14.2	11
	Honduras	8,795	286 (0.8)	14	3.3	14	234 (0.6)	12	2.7	13	1,117 (0.6)	14	12.7	17	874 (0.5)	13	9.9	13
	Paraguay	6,905	121 (0.3)	18	1.8	18	167 (0.4)	15	2.4	14	1,185 (0.7)	13	17.2	14	457 (0.3)	17	6.6	17
	El Salvador	6,551	356 (0.9)	13	5.4	11	144 (0.4)	16	2.2	17	1,022 (0.6)	16	15.6	15	637 (0.4)	15	9.7	14
	Nicaragua	6,361	305 (0.8)	15	4.8	12	153 (0.4)	17	2.4	15	1,117 (0.6)	15	17.6	13	562 (0.3)	16	8.8	15
	Costa Rica	4,949	142 (0.4)	16	2.9	16	201 (0.5)	13	4.1	8	983 (0.6)	17	19.9	12	685 (0.4)	14	13.8	12
	Panama	3,842	127 (0.3)	17	3.3	15	88 (0.2)	18	2.3	16	569 (0.3)	18	14.8	16	278 (0.2)	18	7.2	16
	Uruguay	3,487	383 (1.0)	12	11	2	761 (1.9)	8	21.8	1	1,516 (0.9)	12	43.5	4	1,303 (0.8)	11	37.4	3
Other countries																		
	USA	325,318	673 (1.8)		0.2		1,310 (3.3)		0.4		3,319 (1.9)		1.0		12,166 (7.0)		3.7	
	Brazil	207,012	281 (0.7)		0.1		506 (1.3)		0.2		2,264 (1.3)		1.1		2,124 (1.2)		1.0	
	Portugal	10,265	32 (0.1)		0.3		45 (0.1)		0.4		2,12.0 (0.1)		2.1		406.0 (0.2)		4.0	
	Puerto Rico	3,405	469 (1.2)		13.8		304 (0.8)		8.9		1,495 (0.9)		43.9		815 (0.5)		23.9	

Pop., population; No., number in an ordered list.
